# Inhibiting microcephaly genes as alternative to microtubule targeting agents to treat brain tumors

**DOI:** 10.1038/s41419-021-04259-6

**Published:** 2021-10-18

**Authors:** Giorgia Iegiani, Ferdinando Di Cunto, Gianmarco Pallavicini

**Affiliations:** 1 Neuroscience Institute Cavalieri Ottolenghi, 10043 Orbassano, Italy; 2 Department of Neuroscience ‘Rita Levi Montalcini’, University of Turin, 10126 Turin, Italy

**Keywords:** CNS cancer, Microtubules, Neural progenitors

## Abstract

Medulloblastoma (MB) and gliomas are the most frequent high-grade brain tumors (HGBT) in children and adulthood, respectively. The general treatment for these tumors consists in surgery, followed by radiotherapy and chemotherapy. Despite the improvement in patient survival, these therapies are only partially effective, and many patients still die. In the last decades, microtubules have emerged as interesting molecular targets for HGBT, as various microtubule targeting agents (MTAs) have been developed and tested pre-clinically and clinically with encouraging results. Nevertheless, these treatments produce relevant side effects since they target microtubules in normal as well as in cancerous cells. A possible strategy to overcome this toxicity could be to target proteins that control microtubule dynamics but are required by HGBT cells much more than in normal cell types. The genes mutated in primary hereditary microcephaly (MCPH) are ubiquitously expressed in proliferating cells, but under normal conditions are selectively required during brain development, in neural progenitors. There is evidence that MB and glioma cells share molecular profiles with progenitors of cerebellar granules and of cortical radial glia cells, in which MCPH gene functions are fundamental. Moreover, several studies indicate that MCPH genes are required for HGBT expansion. Among the 25 known MCPH genes, we focus this review on KNL1, ASPM, CENPE, CITK and KIF14, which have been found to control microtubule stability during cell division. We summarize the current knowledge about the molecular basis of their interaction with microtubules. Moreover, we will discuss data that suggest these genes are promising candidates as HGBT-specific targets.

## Facts

- High-grade brain tumors (HGBT) are an unmet medical challenge.
- Microtubule targeting agents (MTAs) have been tested against HGBT but show high toxicity.
- Primary hereditary microcephaly genes like KNL1, ASPM, CENPE, CITK and KIF14 act on microtubules and are required for mitosis mostly in neural precursor cells.
- HGBT share molecular profiles with neural precursor cells.
- Inactivation of KNL1, ASPM, CENPE, CITK and KIF14 leads to mitotic catastrophe, cell cycle arrest and apoptosis in HGBT cells, and may thus represent a more specific therapeutic strategy in alternative to MTAs.

## Open question

- Develop specific inhibitors for KNL1, CITK and ASPM.
- Test in preclinical models CENPE and KIF14 specific inhibitors.
- Test whether the inhibition of these molecules may synergize with standard treatments, as radiation or chemotherapy.

## Introduction

High-grade brain tumors (HGBT) are very aggressive cancers that represent an important unmet medical challenge. Medulloblastoma (MB) is the most common pediatric brain tumor but occurs also in adults. Based on microarray and genomic sequencing technologies, MB has been divided into four biological subgroups (WNT, SHH, Group 3, and Group 4) [1, 2]. MB is currently treated with surgery, followed by irradiation of the entire neuroaxis and high-dose multi-agent chemotherapy. Long-term survival rates can be as high as 90% in the rare WNT subgroup, but they are usually around 50% in most other cases, with an intermediate prognosis in Group 4 and worse in Group 3 patients [3, 4]. Thus, many patients still die despite treatment and those who survive suffer from neurological, cognitive and endocrine disorders caused by the aggressive therapy [3–5]. In adulthood, the most frequent HGBT are gliomas. Among them, glioblastoma multiforme (GBM) is one of the deadliest human cancers. According to WHO, GBM accounts for ~12–15% of all brain tumors, and 60–70% of astrocytic tumors [6]. Standard therapy for GBM is mainly based on surgical resection in combination with radiotherapy and chemotherapy with alkylating agents, such as Temozolomide (TMZ). Gene expression profiling has allowed to classify GBM into four distinct subtypes associated with distinct genomic abnormalities and different responses to aggressive therapy [7]. Nevertheless, the longest median survival obtained in GBM patients treated with combined therapy is 14 months [8] and the 10-year survival rate in the population with GBM is 0.71% [9]. For these reasons, more effective therapies must be developed. On this line, therapies already developed for other types of cancers have been tested for HGBT. Old and new evidence indicates that targeting microtubules could improve outcome in HGBT [10–12].

## Microtubule targeting agents (MTAs) in brain tumors

Microtubules are highly dynamic cytoskeletal components that are essential for many cellular functions such as intracellular organization, ordered vesicle transport, and cell division. The basic building blocks of microtubules are heterodimers of globular α- and β-tubulin subunits, each of which consists of multiple isotypes, differing in amino acid sequence and encoded by different genes [13]. The block of microtubule polymerization dynamics leads to disruption of cellular division, causing mitotic catastrophe, cell cycle arrest, and apoptosis [14]. Therefore, many MTAs have been purified and synthesized to be used as therapy for a variety of cancers [15]. MTAs are often classified into two groups: microtubule-stabilizing agents that increase microtubule polymerization at high concentrations and include paclitaxel, docetaxel, the epothilones and discodermolide [11, 15, 16]; microtubule-destabilizing agents, such as the Vinca alkaloids (vinblastine, vincristine, vinorelbine), combretastatins, estramustine and colchicine, which inhibit microtubule polymerization at high concentrations [11, 15, 16]. At lower concentrations, drugs in both groups suppress microtubule dynamics without changing the microtubule-polymer mass [17]. MTAs exert their effects suppressing the spindle microtubules dynamic, which results in slowdown or block of mitosis. This block, occurring in the G2/M phase, can trigger cell death through apoptosis [16].

When used at low doses, MTAs prevent the proper alignment of chromosomes during metaphase and the correct segregation of chromosomes in anaphase [11]. Moreover, MTAs at low doses can lead to mitotic slippage with G1 arrest [18].

Over the years, several MTAs approved by FDA were tested on MBs, like paclitaxel and docetaxel, obtaining encouraging results [19, 20] (Table 1). Both stabilizing and destabilizing agents have been tested also on gliomas pre-clinically [21–23] and clinically in combination with other treatments [24–28] (Table 1). Using digital modeling algorithms, several new compounds have been discovered: IDN5390111 and other seco-taxanes, such as ixabepilone and patupilone (epothiloneB) [29]. These drugs act by antagonizing the assembly of cytoskeletal microtubules, leading to inhibition of migration and invasion of GBM cells, together with the destabilization of microtubule dynamics in the mitotic spindle [21, 30]. Results from a phase I/II clinical trial which tested patupilone in recurrent GBM, reported that two patients out of nine benefited from long-term recurrence-free survival [31]. As a less toxic alternative to vincristine, patupilone was also tested in preclinical studies on MB [32]. Lastly, sagopilone, an analogue of patupilone, has been tested in 15 patients with recurrent GBM, since it demonstrated promising results in rodent models of GBM [33–35]. All together, these studies have shown that targeting microtubules in HGBT could have beneficial effects. However, side effects induced using MTAs in oncology are the other face of the coin. Indeed, these drugs are inherently nonspecific, as they target microtubules in cancerous and normal cells. Peripheral neuropathies and autonomic neuropathies are commonly observed as side effects, since the neuronal activity is highly dependent on the proper functioning of microtubules [36, 37] (Table 1). Some other adverse effects observed are nausea, dizziness and febrile neutropenia-like septic death [19]. Another side effect is represented by hematological toxicity, which leads to myelosuppression by inhibition of dividing hematopoietic cells [18] (Table 1).

**Table 1:** List of the clinical trials with microtubule targeting agents on HGBT patients.

Compound	Disease	Stage of clinical development	Results	Side effects	References
Paclitaxel	Astrocytoma, Malignant glioma, Medulloblastoma, Brain stem glioma, Ependymoma	Phase II	Paclitaxel resulted in complete or partial response rate in 5.7% of the patients and stable disease in 14% of the patients. It can be administered with radiotherapy	Hematologic toxicity, febrile neutropenia, nausea and vomiting	[19, 26]
Docetaxel	Astrocytoma, Glioma, Medulloblastoma, Brain stem glioma, Ependymoma	Phase II	Docetaxel was tolerated well but poor effective for treating these types of recurrent solid tumors	Hematologic toxicity and liver and gastrointestinal toxicity in some patients	[20]
Vincristine	Astrocytoma, Oligoastrocytoma, Oligodendroglioma, Glioblastoma	Phase II/III	Combination chemotherapy with Vincristine in addition to radiation therapy resulted in longer overall survival compared to radiation alone	Hematologic toxicity, fatigue, anorexia, nausea, vomiting	[24, 25, 27]
Patupilone (Epothilone B)	Glioblastoma	Phase I/II	Epothilone B was well tolerated and progression free at 6 months was observed in 22% of the patients. It can be administered with radiotherapy	Reversible sensory neuropathy, diarrhea, nausea and vomiting	[28, 31, 32]
Sagopilone	Gliosarcoma, glioblastoma multiforme, anaplastic astrocytoma	Phase II	Progression free at 6 months was observed in 33% of the patients	Peripheral neuropathy, hematologic toxicity	[33–35]

### Inhibition of microcephaly genes as an alternative to direct microtubule targeting

The targeting of proteins that act on microtubules dynamics, but are necessary only for brain cancer cells, could be a specific goal for HGBT treatment. To this regard, an interesting group of potential candidate genes exists, whose products are normally required for the proliferation of neural progenitor cells (NPC), but are much less critical in other cell types. Mutation of these genes results in rare genetic disorders, characterized by a strong reduction of brain volume, referred to as congenital microcephaly (CM). Genes mutated in congenital CM syndromes have already been proposed as possible targets for HGBT-directed drug development [38–40]. Although it is still debated from which NPC different cancers originate, MB and GBM share many molecular features with cerebellar granules progenitors and cortical radial glia cells, respectively [41–44]. Loss of genes associated with CM leads to specific alterations of proliferation and survival of such cells.

CM is a heterogeneous group of disorders characterized by reduced head circumference at birth, to at least 3 standard deviations (SD) below the mean [45]. The simplest form of genetic CM is primary hereditary microcephaly (MCPH), in which brain size reduction is accompanied by normal brain structure and mild intellectual disability [45, 46]. The common feature of MCPH genes is that they are selectively required for proliferation and genomic stability of central nervous system (CNS) cells, despite being expressed in all proliferating cell types [47]. The biological basis of this specificity is only partially understood [46]. Many MCPH proteins are associated to centrosomes and their loss leads to cell cycle and mitosis delay, mitotic failure, and randomization of spindle orientation [48]. These alterations tilt the balance between symmetric and asymmetric divisions of neural stem cells, decreasing the number of proliferating NPCs [48]. However, it has also been reported that MCPH genes loss leads to DNA damage accumulation and apoptosis [49–51]. Despite it is not known the precise mechanisms of these specific vulnerabilities, inactivation of MCPH genes may reduce the expansion of brain tumors.

**Fig. 1:**
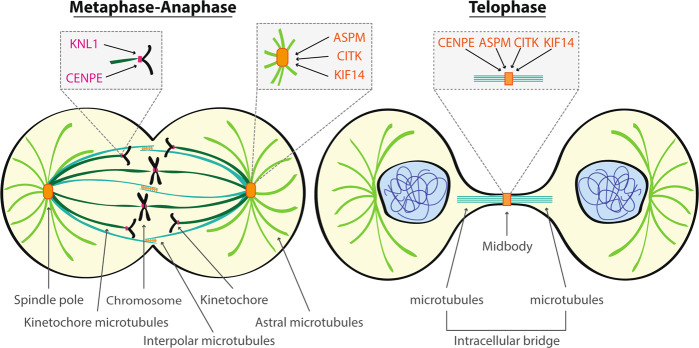
Scheme of KNL1, ASPM, CENPE, CITK, and KIF14 localization during mitosis. During metaphase, KNL1 and CENPE are localized to kinetochore, where they promote microtubule-kinetochore attachment and correct chromosome alignment. KNL1 and CENPE remain localized to kinetochore up to anaphase. During metaphase-anaphase transition, ASPM, CITK, and KIF14 are found at spindle poles and their associated microtubules. ASPM and CITK are able to interact with astral microtubules and promote their growth. In the transition to telophase, CENPE ASPM, CITK, and KIF14 translocate to the central spindle and then localize at the midbody.

Between the 25 genes identified leading MCPH, a subset of them exerts their function principally by altering microtubule dynamics during mitosis. In this review, we focus on those that look like the most promising ones, which are KNL1, ASPM, CENPE, CITK, and KIF14. They are expressed ubiquitously during the cell cycle in most normal cells (Fig. 1) but are specifically necessary for the proliferation of NPC and affect HGBT cells expansion. Inhibition of their function could mimic the action of MTAs, without affecting CNS post-mitotic cells, as well as proliferating cells throughout the body. We highlight what is already known about their molecular action and antineoplastic effects on brain tumors, to suggest a new possible strategy for HGBT therapy.

### KNL1

Kinetochore scaffold 1 (KNL1) gene, also known as CASC5, is located on chromosome 15q15. Recessive mutations in this gene lead to MCPH4 syndrome. Few missense and frameshift mutations have been identified on KNL1 gene in 19 individuals belonging to 7 families [52–55]. All of these mutations cause protein truncation or nonsense-mediated mRNA decay, leading to loss of KNL1 function [52–55]. Individuals affected by MCPH4 are characterized by a reduction of head circumference of 4–7 SD below the mean and impaired cognitive functions [52–55].

KNL1 is ubiquitously expressed, with high expression in fetal tissue and some adult tissues as testis, thymus, and bone marrow [56]. Even though it is expressed in various tissues, it is specifically necessary in NPC. Indeed, patient-derived lymphoblasts and fibroblasts do not show any abnormalities in mitosis and growth rate [53]. Instead, NPCs show reduced cell growth with altered cell-cycle phases and increased cell death [57]. Moreover, these cells show aneuploidy and abrogated spindle assembly checkpoint (SAC) with premature differentiation [57].

### KNL1 controls microtubule-kinetochore attachment

**Fig. 2:**
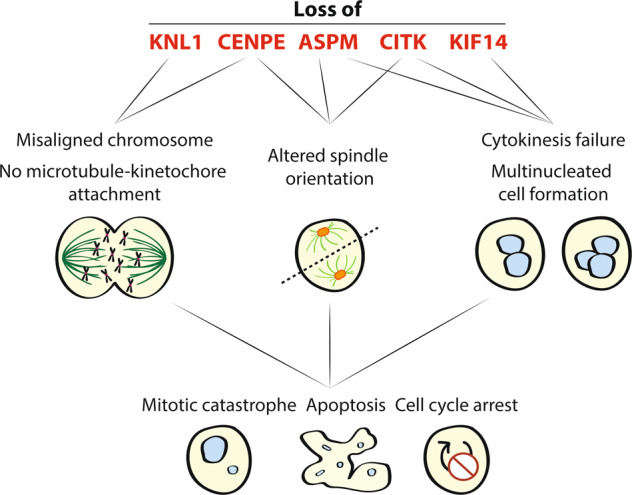
Scheme of the effects observed after KNL1, ASPM, CENPE, CITK, and KIF14 loss. The loss of KNL1 and CENPE expression in proliferating cells leads to misaligned chromosomes and altered microtubule-kinetochore attachment. CENPE, ASPM, and CITK loss determines altered spindle poles orientation and oblique divisions. ASPM, CITK, and KIF14 loss leads to cytokinesis failure with the formation of multinucleated cells. These alterations can result in mitotic catastrophe or apoptosis or cell cycle arrest.

KNL1 is part of the KNL-1/Mis12/Ndc80 complex (KMN) network and is a conserved scaffold protein used for proper kinetochore assembly, checkpoint functioning and SAC signaling. SAC is a safeguard system that prevents the separation of sister chromatids until each kinetochore is properly attached to the spindle poles [58]. Indeed, KNL1 is localized to kinetochores from prophase to early telophase, thanks to the kinetochore localization domain in the C-terminus [59] (Fig. 1). Knockdown of KNL1 leads to SAC failure, mitosis acceleration with frequent chromosome misalignment, impairment of microtubule attachment to kinetochore, micronuclei, and multinucleated cell formation [59, 60] (Fig. 2). The N-terminal domain of KNL1 contains two distinct microtubule-binding regions [61]. Mutations that affect KNL-1 microtubule binding at the N-terminus do not affect chromosome segregation but lead to a significantly increased cell-cycle delay and an extended anaphase duration in the presence of bipolar spindles [62]. Thus, it has been proposed that KNL1 microtubule-binding site senses the presence of microtubules attached to the kinetochore, probably via the Ndc80 complex, and relays their presence to shut off generation of the checkpoint signal [62]. All together, these data indicate that KNL1 is required for correct chromosome segregation via microtubule binding and interaction with the other kinetochore-associated proteins.

### KNL1 is a potential target for brain tumor treatment

KNL1 loss can reduce proliferation, trigger cell cycle arrest and apoptosis in TP53-wt and TP53 mutated cell lines, as well as in vivo in xenograft tumor models [63]. Indeed, the analysis of 17 patient-derived cell lines, including 3 anaplastic oligodendrogliomas, 1 anaplastic astrocytoma and 13 GBMs, found KNL1 highly expressed in all of them, with levels 10-fold higher than in normal brain [64]. Moreover, KNL1 knockdown decreased the proliferation and clonogenic ability of GBM cell line [65] (Table 2). All together, these data indicate that KNL1 depletion could be tested in other HGBT models, to be proposed as a potential new specific target.

**Table 2:** Summary of the molecular action of KNL1, ASPM, CENPE, CITK, and KIF14 on microtubules and their antineoplastic effect on HGBT.

Microcephaly syndrome	Gene name	Protein	Action on microtubules	Ref.	Mitotic effects after loss	Ref.	Antineoplastic effects on HGBT	Ref.
MCPH 4	KNL1	Kinetochore scaffold 1	Binds microtubules at the kinetochore by two distinct microtubule-binding regions at N-terminal	[61]	Chromosome misalignment, no microtubule-kinetochore attachment, and multinucleated cell formation	[59, 60]	KNL1 knockdown decreases the proliferation and cloning ability of GBM cells	[65]
MCPH 5	ASPM	Abnormal spindle-like, microcephaly-associated protein	Controls the number and length of astral microtubules and microtubule disassembly at spindle poles	[71, 72]	Altered spindle orientation, cytokinesis failure, apoptosis	[67, 70, 71]	ASPM loss impairs GBM and MB tumor growth and increases DNA damage	[50, 77–80]
MCPH 13	CENPE	Centromere-associated protein E	Microtubule motor protein. Promotes microtubule elongation at kinetochore and controls the length of astral microtubules	[98–103]	Chromosome misalignment, no microtubule-kinetochore attachment, altered spindle orientation, mitotic arrest	[92–94]	CENPE inhibition reduces proliferation of GBM and MB cells, increases DNA damage and induces mitotic catastrophe	[105, 106]
MCPH 17	CIT	Citron Rho-interacting serine/threonine kinase	Controls the number and length of astral microtubules and promotes microtubule stabilization at midbody	[71, 124]	Altered spindle orientation, cytokinesis failure, apoptosis	[120, 126]	CITK loss impairs MB tumor growth, increases DNA damage and the sensitivity to radiation and cisplatin	[128, 129]
MCPH 20	KIF14	Kinesin like protein 14	Microtubule motor protein with ATP biding motif, promotes microtubule stabilization	[137]	Cytokinesis failure, apoptosis	[136]	KIF14 knockdown reduces proliferation of GBM and MB and induces apoptosis	[105, 139, 140]

### ASPM

Abnormal spindle-like microcephaly-associated (ASPM) gene is located on chromosome 1q31. Recessive mutations, identified so far in 638 individuals belonging to 282 families, cause the MCPH5 syndrome [66]. The majority of these mutations lead to protein truncation and include exonic nucleotide substitutions, deletions and intronic variations [66]. Individuals affected by this disease are characterized by a decreased occipitofrontal circumference of 3–11 SD below the mean [66]. ASPM is expressed in both fetal and adult tissues, including the heart, liver, skeletal muscle, and lung [67]. Although it is expressed in various tissues, it is most important in the brain at the cortical ventricular zone and in the proliferation zones of the medial and lateral ganglionic eminence [68, 69]. In these areas, the expression of ASPM is greatly reduced by the day of birth when neurogenesis is mostly completed [68, 69]. Instead, in the cerebellum, ASPM is also required postnatally, during cerebellum development [50].

### ASPM regulates spindle formation stabilizing microtubules

ASPM exerts its function during the cell cycle: it is predominantly nuclear in interphase cells and localizes to the spindle poles during metaphase, around γ-tubulin cluster [67]. During telophase, ASPM is localized to the minus ends of central spindle microtubules and in late telophase at the midzone of the central spindle [67, 70]. Finally, ASPM is located at the midbody during cytokinesis [67, 70] (Fig. 1). Loss of ASPM induces misorientation of the cell division plane and mitotic spindle, cytokinesis failure with the formation of binucleated and multinucleated cells, as well as increased apoptosis [67, 70, 71] (Fig. 2). Moreover, ASPM depletion leads to a reduction in the number and length of astral microtubules [71]. Combination of ASPM knockdown with the microtubule-stabilizing drug paclitaxel, was shown to restore the mitotic spindle angles to control values, indicating that ASPM alone can stabilize microtubules [71]. Lastly, ASPM is able to control microtubule disassembly at the spindle poles and inhibit microtubule minus ends growth together with KATNA1, a conserved microtubule-severing protein [72]. To sum up, ASPM plays a critical role in spindle microtubule organization, spindle positioning, and in the regulation of cytokinesis in both neural and non-neural cells [67].

### ASPM loss reduces growth and increases apoptosis of GBM and MB

Different studies have shown that ASPM expression correlates with WHO grade of the astrocytic tumors, being higher in glioblastomas and in recurrent tumors [73–75]. Moreover, a recent study, that analyzed two glioma databases, identified ASPM as one of the 10 hub genes most associated with carcinogenesis and the development of GBM [76]. In different GBM cell lines and patient-derived GBM cells, ASPM knockdown by siRNA reduces proliferation, increases cell death, impairs DNA double-strand break repair, and enhances the sensitivity to X-rays [77]. ASPM knockdown increases chromosome aberrations in irradiated cells and inhibits homologous recombination [78] and non-homologous end joining (NHEJ) pathway in GBM [77]. Stable ASPM knockdown results in cell cycle arrest in G0/G1 phase [79, 80]. In vivo, ASPM’s high expression was shown to enhance the tumorigenicity of GBM xenograft model [79] and its depletion resulted in reduced tumor growth [80]. With regard to pediatric brain tumors, ASPM was found highly expressed in MB samples, compared to normal tissue, and its levels correlated with worse overall survival of MB patients [81, 82]. In a primary mouse model of MB, conditional ASPM deletion impairs tumor growth, increases DNA damage, and reduces hydrocephalus [50]. In conclusion, since ASPM is involved in microtubule stabilization, its depletion could mimic microtubule-destabilizing agents, with less side effects on other CNS cell types (Table 2). Unfortunately, an ASPM specific inhibitor has not been proposed yet. A screening by qRT-PCR identified six compounds from a library of 31,624 small molecules that decreased ASPM RNA levels [83] but validation of antineoplastic effects in brain tumors is still missing.

### CENPE

Centromere-associated protein E (CENPE) gene is located on chromosome 4q24. CENPE heterozygous mutations have been identified only in two siblings, a boy, and a girl, and cause the MCPH13 syndrome [84]. Both showed a reduction of head circumference of 7–9 SD below the mean, sloping forehead, large ears, and nose. No mice carrying homozygous CENPE mutations have been described, consistent with the fact that in humans only heterozygous mutations have been found and suggesting a strong embryonic requirement of full gene dosage. Similar to the other microcephaly genes, CENPE is expressed in various tissues including lymph-node, testis, bone marrow, appendix, and brain [85].

### CENPE controls chromosome-microtubule attachment through microtubule stabilization

CENPE is a microtubule plus-end-directed kinetochore motor protein important in chromosome congression, spindle microtubule capture at kinetochores, and SAC activation [86]. It is expressed during the cell cycle, reaching its peak during G2/M phase [87]. CENPE is not present during interphase and appears at the centromere region of chromosomes during prometaphase [88]. It is localized to the kinetochore of chromosomes where it controls chromosome alignment by capturing microtubule plus ends at the kinetochore [88–90]. Indeed, during cell division, CENPE interacts with the mitotic centromere-associated kinesin (MCAK) to regulate chromosome-microtubule end-on attachment [86]. During the transition to anaphase, CENPE translocates to the central spindle and then is localized at the central region of midbody during cytokinesis [91] (Fig. 1). Disruption of CENPE prevents chromosome alignment, inhibits microtubules attachment to kinetochores, and induces mitotic arrest [92–94] (Fig. 2). In cells transfected with CENPE siRNA, the majority of bipolar spindles were blocked at prometaphase and metaphase. Moreover, cells displayed a long delay in the metaphase-to-anaphase transition [95]. No effects on mitotic spindle assembly were observed [96]. CENPE possesses at the C-terminus a kinetochore-binding domain that allows the interaction with kinetochore proteins BUBR1, CENPF, Ndc80, and HsNUF2 [86, 97]. Instead, an ATP-dependent microtubule-binding site is present at the N-terminus, which is used for hydrolyzing ATP to generate mechanical forces along microtubules and is essential for CENPE localization at mitotic spindles [98]. In particular, CENPE moves toward the plus end of MT and is able, together with HsNUF2, to stabilize the association between microtubule and kinetochore [98, 99]. Moreover, CENPE promotes microtubule elongation at kinetochore and stabilize its conformation. This is mediated by ATP turnover [100]. The motor activity of CENPE and motif in the C-terminal domain are also involved in anchoring CENPE to the center of the midbody [101]. Lastly, loss of CENPE induces shortened astral microtubule and oblique cell divisions [102, 103]. All together these data illustrate the important role that CENPE plays during the cell cycle which is mediated by its microtubule-binding activity.

### CENPE loss reduces cell proliferation in GBM and MB

The analysis of four Affimetrix GeneChip datasets, comprising 771 glioma samples, revealed that CENPE expression correlates with WHO grade of glioma patients, with a higher expression in grade IV tumors [104]. In pediatric high-grade glioma cell lines, CENPE knockdown, alone or in combination with TZM, reduces cell proliferation and induces cell cycle arrest [105]. Moreover, CENPE knockdown induces mitotic catastrophe and DNA damage in MB cell lines [106]. All together these data suggest that CENPE could be a potential target in GBM and MB treatment, blocking microtubule stabilization and impairing mitosis (Table 2). Interestingly, different molecules have been proposed as CENPE inhibitor. Syntelin was shown to inhibit CENPE motor domain [107]. Another inhibitor, PF-2771, is a specific non-competitive molecular inhibitor tested against triple-negative breast cancer model [108]. Compound-A was found to inhibit CENPE activity through the competition with its ATPase pocket of CENPE motor domain in HeLa cells [109]. Lastly, the allosteric inhibitor GSK923295 binds the ATPase pocket and has already finished the phase I clinical trial [110]. This compound was shown to be effective against MB cell lines, where it abolished the clonogenic potential and induced mitotic catastrophe and DNA damage [106]. Given this result in MB, it would be interesting to test the efficacy of this compound in other in vitro and in vivo HGBT models.

### CITK

Citron Kinase (CITK) is the major product of the CIT gene, located on chromosome 12q24. Homozygous or compound heterozygous mutations in the CITK gene cause the MCPH17 syndrome, identified so far in 20 people belonging to 9 different families [111–114]. Notably, some patients presented the mutations in the kinase domain of CITK protein, determining loss of catalytic activity [114]. Individuals affected by MCPH17 showed very small head circumference apparent at birth that worsens over time up to 8 SD below the mean. In these patients, skin-derived fibroblasts do not show any defects in cell proliferation or mitosis [114]. Instead, NPCs show cytokinesis failure, with the formation of binucleated cells, multipolar spindles, and apoptotic cells [114]. CITK is expressed in all proliferating cells during the cell cycle [115], although it is functionally required in NPCs, as previously demonstrated in preclinical models [116, 117], and in spermatogenic precursors [118].

### CITK regulates different stages of mitosis and cytokinesis, by stabilizing microtubules

CITK reaches its peak of expression during the G2/M phase of the cell cycle. It is nuclear during interphase, cytoplasmic at early prophase, enriched at spindle poles before anaphase, and is localized at the cleavage furrow and at midbody during cytokinesis [115, 119] (Fig. 1). Loss of CITK leads to cytokinesis failure with the formation of binucleated and multinucleated cells [120]. Moreover, the absence of CITK does not alter cleavage furrow formation, cleavage furrow ingression or midbody formation, but leads to midbody instability (Fig. 2). Indeed, CITK is required for the correct localization of various proteins at midbody including RhoA, Anillin, Aurora B, and KIFBP [120–125]. It has also been demonstrated that CITK is required for midbody late-stage maturation and for the final cut of cell bridge [126]. In the complex network of proteins that regulate cytokinesis, CITK function is strictly related to microtubule organization. CITK recruits at the midbody the kinesins KIF14 and KIF23, which in turn recruit the microtubule-cross-linking protein PRC1 [127]. Moreover, CITK knockdown alters the ratio between tyrosinated and acetylated tubulin and increases microtubule turnover at midbody [124]. It may stabilize midbody microtubules via casein kinase 2 and consequent phosphorylation of TUBB3 [124]. Combination of CITK knockdown with microtubule-destabilizing agent (nocodazole) increased the percentage of binucleated HeLa cells [124]. Instead, the combination of CITK knockdown with a stabilizing agent (paclitaxel) reduced cytokinesis failure and restored the mitotic spindle angles to control values [71, 124]. Lastly, CITK depleted cells show a reduction in the number and length of astral microtubules and decrease microtubule stability and nucleation in mitotic cells [71]. These findings indicate that CITK is involved in microtubule stabilization during metaphase and intercellular bridge formation.

### CITK loss induces apoptosis and cell cycle arrest in MB

It has been demonstrated that CITK could be a potential target for medulloblastoma treatment [128, 129]. CITK knockdown by RNAi in SHH, Group 3 and 4 medulloblastoma cell lines impairs proliferation, induces cytokinesis failure, cell cycle arrest, and apoptosis via TP53-dependent and TP53 independent mechanisms [128–130]. Interestingly, in CITK-depleted cells, overexpression of CITK protein mutated in the kinase domain is not able to rescue proliferation [128]. Moreover, all CITK-depleted cell lines show an accumulation of DNA damage, consistent with data obtained in neural progenitors of null mice [49]. Interestingly, these cells show also reduced level of the DNA-repair protein RAD51 and impairment of homologous recombination [129]. Lastly, CITK knockdown in MB cells potentiates the effect of radiation and cisplatin treatment [129]. In vivo, CITK depletion limits the growth of xenograft tumors as well as of tumors arising in the transgenic MB model and in the latter case also increases survival [128]. The cytokinesis failure and cell cycle arrest observed in MB cells after CITK knockdown could be associated with its activity on microtubule dynamics (Table 2). On this basis, developing a specific inhibitor for CITK could be interesting for HGBT targeted therapy.

### KIF14

KIF14 gene is located on chromosome 1q31-1q32 and encodes for a member of the kinesin-3 superfamily of microtubule motor proteins. Recessive mutations in this gene cause the MCPH20 syndrome. This syndrome has been described in 13 different families, in which missense, nonsense, and frameshift mutations have been found [131–133]. Individuals affected by this disease are characterized by a decreased occipitofrontal circumference of 3.6–11 SD below the mean, intellectual disability, variable speech impairment, and developmental delays [131–133]. KIF14 is expressed at low levels in normal adult tissues and at higher levels in placenta and fetal tissues, with the highest expression in fetal thymus and liver [134]. In particular, KIF14 is fundamental for brain development: a fetus with KIF14 mutations presented microcephaly with a flattened forehead, strong delay in the development of the telencephalon, and hypoplasia of the cerebellum [133].

### KIF14 is essential for late stages of cytokinesis and intercellular bridge cut

KIF14 is required during the cell cycle and reaches its peak in the G2/M phase [135]. It is located in the cytoplasm during interphase; during prophase and metaphase accumulates at the developing spindle poles and their associated microtubules [135] (Fig. 1). During anaphase KIF14 localizes to the spindle midzone, whereas during telophase it is concentrated at the midbody [135]. Finally, during cytokinesis, KIF14 is located at the contractile ring where it localizes with CITK [136] (Fig. 1). KIF14 knockdown does not alter chromosome congression and alignment but it induces cytokinesis failure [135]. KIF14-depleted cells segregate chromosomes, proceed through anaphase, initiate furrow formation and elongate the midbody, but fail to cleave the intracellular bridge, resulting in midbody collapse and increase the rate of binucleated cells [136] (Fig. 2). Consistent with its function as a microtubule motor protein, KIF14 possesses an internal motor domain with robust ATPase activity and high affinity for microtubules [137]. KIF14 is an extremely slow and inefficient walking motor, but its motor domain is able to protect microtubules from cold-induced depolymerization [137]. Mutations in the motor domain severely impair microtubule binding, making the kinesin not functional [133]. These data indicate that KIF14 plays an important physiological role during cell division and that the ability to bind microtubules is essential for its function.

### KIF14 reduces tumor growth and induces apoptosis in HGBT

KIF14 was proposed as prognostic marker for glioma patients [138]. In 20 glioma tissues, KIF14 levels were increased compared to non-neoplastic brain tissues and they correlated with the tumor pathological grade, being higher in grade II–IV [138]. High KIF14 levels were associated with a higher mitotic and Ki67 index, as well as with a lower patient survival rate [138]. Transient KIF14 depletion decreases GBM cell growth, induces an accumulation of cells in G2/M phase and increases the levels of binucleated and apoptotic cells, accompanied by inactivation of Akt kinase [139]. Moreover, combination of stable KIF14 knockdown with TMZ synergize in reducing the proliferation of pediatric high-grade glioma cell lines [105]. In vivo, KIF14 depletion was shown to reduce tumor growth in GBM xenograft model [139]. KIF14 was found more expressed in various MB cell lines compared to the normal fetal brain [134] and in primary tumors, in which its levels correlated with poor survival [140]. Stable KIF14 knockdown in MB cell lines reduces cell proliferation, induces apoptosis, impairs clonogenic capacity, and reduces cell migration and invasion potential [140]. All together these data indicate that KIF14 could be an interesting target for HGBT treatment (Table 2). A putative KIF14 inhibitor, identified in a screen of small molecules that selectively inhibit its ATPase activity, was tested against three different triple-negative breast cancer cell lines [141]. It will be interesting to validate it on HGBT pre-clinical models.

## Conclusions

HGBT are very aggressive cancers with poor prognosis and represent an important unmet medical challenge. In recent years, innovative therapies have been developed, which could be associated with standard treatments. To develop new treatment schemes, MTAs have been tested for HGBT. Blocking microtubule dynamics leads to disruption of cellular division, cell cycle arrest, and apoptosis. Despite encouraging results on patients, heavy side effects are present.

Targeting MCPH genes that act on microtubule dynamics could represent a valuable alternative to MTAs. Among them, all five proteins presented in this review play a role during the cell cycle, in the G2/M phase, and are localized to kinetochore, spindle poles or midbody (Fig. 1). All of them can stabilize microtubules to which they are associated to. Their downregulation leads to chromosome instability, spindle mispositioning, and cytokinesis failure, resulting in mitotic catastrophe, cell cycle arrest, and apoptosis (Table 2 and Fig. 2). Since it has been observed that altering microtubule dynamics disrupts the trafficking of DNA repair proteins [142], this mechanism could be responsible not only for the cell division defects, but also for the DNA damage caused by MCPH loss. MTAs do not act specifically and can target microtubules also in normal cells. In contrast, targeting MCPH genes, which are mostly required for mitosis of NPC, would lead to specific microtubules destabilization in brain tumor cells.

The fact that KNL1, CENPE, ASPM, and KIF14 show high expression in GBM and MB samples supports this possibility. Additional support derives from the finding that these genes may show a lower than expected frequency of inactivating mutations in cancer [143], indicating the existence of a selective pressure against their loss. Results obtained in preclinical models of GBM and MB are consistent with these considerations. This is especially evident for ASPM and CITK, since total body depletion of these proteins in MB mouse model didn’t lead to measurable physiological alteration in other organs [116, 144].

It is unlikely that inhibitory strategies targeted to these proteins could be used alone in cancer treatment. Therefore, it will be especially important to test whether they are capable of synergizing with standard treatments, such as radiation or DNA-damaging chemotherapy. In this context, ASPM downregulation was shown to potentiate the effects of X-rays on GBM cells [77], while CITK loss was shown to enhance the effects of X-rays and cisplatin on MB cells [129]. CENPE and KIF14 downregulation was shown to potentiate the effects of TZM in reducing glioma cell proliferation [105].

Potential therapeutic strategies affecting all the proposed genes are in different stages of pre-clinical development. In the case of KNL1 and ASPM, the most promising strategy seems the identification of compounds that may decrease RNA levels [83]. CITK is a druggable protein since it is a serine/threonine kinase, but it is still orphan of a specific inhibitor. A molecule that selectively inhibits KIF14 ATPase activity was identified in a screen of small molecules [141]. Ultimately, a plethora of different molecules have been proposed as CENPE inhibitor: Syntelin, PF-2771, and Compound-A were shown to inhibit CENPE motor domain [107–109]; but the most promising molecule is GSK923295 that has already finished phase I clinical trial for other types of cancers [110] and it was shown to be effective in MB [106]. In conclusion, inhibition of KNL1, CENPE, ASPM, CITK, and KIF14 may mimic the effects of MTAs on cell division but could work with particular effectiveness on brain tumor cells, underscoring the importance of developing specific inhibitors and test them in clinical trials.
